# Adult Murine Skeletal Muscle Contains Cells That Can Differentiate into Beating Cardiomyocytes In Vitro

**DOI:** 10.1371/journal.pbio.0030087

**Published:** 2005-03-15

**Authors:** Steve O Winitsky, Thiru V Gopal, Shahin Hassanzadeh, Hiroshi Takahashi, Divina Gryder, Michael A Rogawski, Kazuyo Takeda, Zu X Yu, Yu H Xu, Neal D Epstein

**Affiliations:** **1**Molecular Physiology Section, Laboratory of Molecular CardiologyNational Heart, Lung, and Blood Institute, Bethesda, MarylandUnited States of America; **2**Epilepsy Research Section, National Institute of Neurological Disorders and StrokeBethesda, MarylandUnited States of America; **3**Laboratory of Molecular Cardiology, National HeartLung, and Blood Institute, Bethesda, MarylandUnited States of America; **4**Pathology Section, National HeartLung, and Blood Institute, Bethesda, MarylandUnited States of America; **5**Electron Microscopy Core Facility, National HeartLung, and Blood Institute, Bethesda, MarylandUnited States of America; University of California at San DiegoUnited States of America

## Abstract

It has long been held as scientific fact that soon after birth, cardiomyocytes cease dividing, thus explaining the limited restoration of cardiac function after a heart attack. Recent demonstrations of cardiac myocyte differentiation observed in vitro or after in vivo transplantation of adult stem cells from blood, fat, skeletal muscle, or heart have challenged this view. Analysis of these studies has been complicated by the large disparity in the magnitude of effects seen by different groups and obscured by the recently appreciated process of in vivo stem-cell fusion. We now show a novel population of nonsatellite cells in adult murine skeletal muscle that progress under standard primary cell-culture conditions to autonomously beating cardiomyocytes. Their differentiation into beating cardiomyocytes is characterized here by video microscopy, confocal-detected calcium transients, electron microscopy, immunofluorescent cardiac-specific markers, and single-cell patch recordings of cardiac action potentials. Within 2 d after tail-vein injection of these marked cells into a mouse model of acute infarction, the marked cells are visible in the heart. By 6 d they begin to differentiate without fusing to recipient cardiac cells. Three months later, the tagged cells are visible as striated heart muscle restricted to the region of the cardiac infarct.

## Abstract 2

A population of primitive cells from adult murine skeletal muscle can develop into beating cardiomyocytes in vitro and can contribute to the repair of damaged heart in vivo

## Introduction

The difficulty in recovery of cardiac function after cardiomyocyte death, such as occurs with a heart attack, contrasts with injury to skeletal muscle in which myocyte numbers can increase through the recruitment of new myocytes from a local stem-cell pool called satellite cells. At present, cardiac transplant, with the intrinsic limitations of supply, immunosuppression, and organ rejection, remains the only long-term treatment for irreversible cardiac failure. Injection of fetal or embryonic stem cells into infarcted hearts holds some promise [1,2,3] but is complicated by the potential for immunologic rejection as well as by political and ethical considerations. Cell plasticity observed after in vivo transplantation of a variety of cell lineages [4,5,6,7] or after in vitro transformation with 5-azacytidine [8] has encouraged the study of cell-based therapy. Investigators have identified endogenous cardiomyocyte proliferation [9] and have experimented with skeletal myoblasts as well as with adult stem cells isolated from blood or heart to try to repair cardiac damage [9,10,11,12,13,14,15].

In vivo analysis of stem-cell transplantation studies has become complicated in part because of a growing understanding of the process of in vivo donor stem-cell and recipient mature-cell fusion [16,17,18]. Recent studies have shown a small population of cells in murine fat that progress to beating cells in vitro [19]. Three independent groups have isolated stem cells from the heart that show a capacity to differentiate into cardiomyocytes in vivo [14,15,20]. All three cell types are extensively passaged, after which two of these can be pushed to differentiate in vitro.

In evaluating a variety of primary cell cultures of different lineages, we noticed nonadherent cells isolated from adult murine skeletal muscle that become beating floating cells. We named them “Spoc” cells (skeletal-based precursors of cardiomyocytes) for their ability to generate beating cardiomyocytes in vitro. These cells, easily obtained from skeletal muscle, provide new options for cardiac research and therapeutic interventions. When injected systemically into an acutely infarcted mouse, they migrate to the cardiac infarct region and differentiate into cardiac myocytes.

## Results/Discussion

### Characterization of Spoc Cells

**Figure 1:**
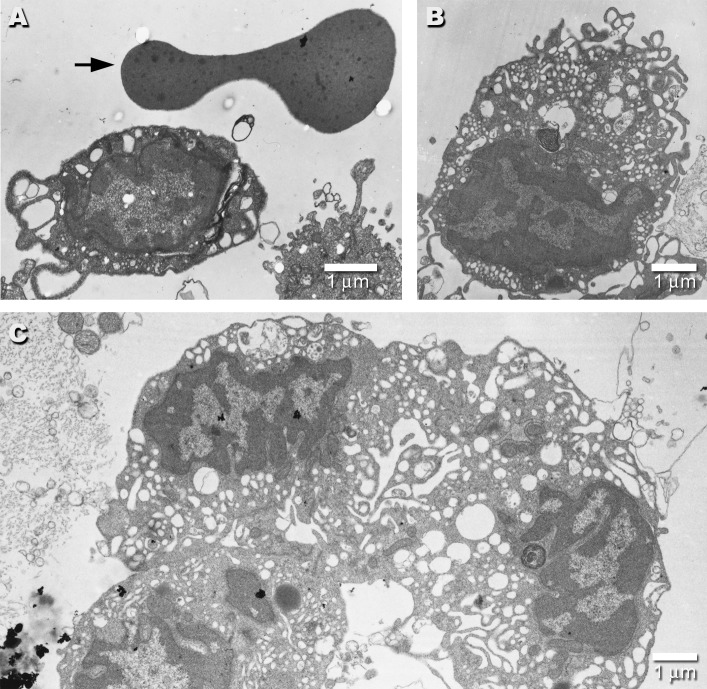
EMs of Day 0 Spoc Cells Electron micrograph of a freshly isolated Spoc cell (A) shows its small size relative to the typical biconcave profile of the red blood cell above it (arrow). (B) shows a typical Spoc cell with a large number of vesicular structures, lamellipodia, and filopodia. (C) displays three Spoc cells so tightly clumped that their borders are difficult to distinguish. This typical clumping makes fluorescence-activated cell sorting (FACS) analysis difficult.

At initial isolation, Spoc cells are CD34^−^ and CD45^−^ (data not shown). Dissociation of 10 g of leg muscle yields approximately 2 × 10^6^ Spoc cells. C-kit^+^ cells, comprising less than 1% of Spoc cells, are present after isolation but can be removed by affinity purification without any change in the subsequent results (data not shown). Electron microscopy (EM) shows that on the day of isolation, Spoc cells are 4–8 μm in diameter, often smaller than a red blood cell, and display copious vesicles suggestive of active transport. Many lamellipodia and filopodia are present, which may in part explain the typical Velcro-like clumping of the cells that makes chemical and physical dispersion more difficult (Figure 1). The CD34^−^/CD45^−^/c-kit^−^ phenotype differentiates Spoc cells from other nonadherent cells previously described as derived from skeletal muscle [5]. Spoc cells are distinguished from satellite cells by the following criteria: (1) Spoc cells do not express Pax-7 [21] or the surface marker c-met [22]; (2) approximately the same number of Spoc cells are isolated from both young (less than 4-wk-old) and older (12- to 16-wk-old) mice, whereas satellite cells are difficult to isolate from normal mouse muscle after 8 wk of age without first inducing muscle injury [21]; (3) at isolation, Spoc cells remain round floating cells approximately 4 μm in diameter, whereas isolated satellite cells soon become adherent; (4) Spoc cells are CD34^−^ and Myf-5^−^, excluding them from the class of quiescent satellite cells [23]; and (5) the three skeletal muscle super-regulatory genes *myf5, myoD,* and *myogenin* are known to be present at some time in satellite cells [22], but in Spoc cells, all three markers remain negative from the day of isolation throughout long-term culture (data not shown). Thus, Spoc cells are neither satellite cells nor further-differentiated skeletal muscle cells.

**Figure 2:**
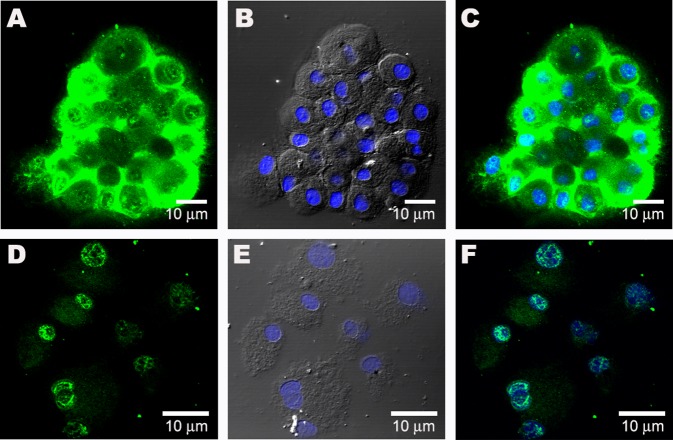
Sublocalization of GATA-4 in Spoc Cells (A) GATA-4 is detected in the cytoplasm of day 10 Spoc cells (cytospin). (B) Nomarski image of (A), showing DAPI-stained blue nuclei. (C) Merge image showing nuclei and GATA-4 staining. (D) When Spoc cells are incubated with 20 μM isoproterenol for 1 h, the GATA-4 nuclear staining is seen. (E) Nomarski image of (D). (F) Merge of (D) and (E), showing sublocalization of GATA-4 to nuclei. A weaker GATA-4 signal is present in the cytoplasm.

**Figure 3:**
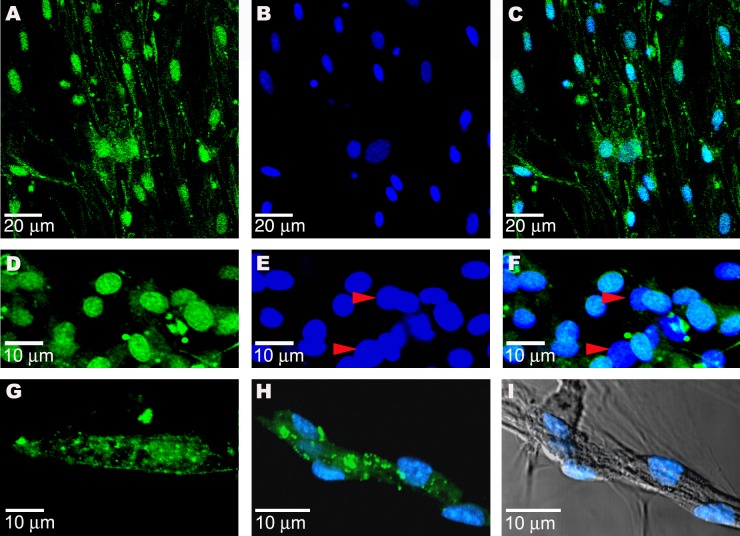
CPS Cells Stain Positive for Cardiac-Specific Proteins (A) GATA-4 in day 7 CPS cells. (B) Nuclear staining with DAPI. (C) Overlay of (A) and (B). (D) Nkx-2.5 is detected in the nuclei of round, day 21 beating cells (green). (E) Noncardiac cells (red arrowheads) do not show nuclear staining for Nkx-2.5. (F) Overlay of (D) and (E). (G) Beating cells, after 28 d in culture, stain positive for cardiac L-type Ca^++^ channel. (H) Connexin 43 (green) in cluster of uninucleate day 21 beating cells in culture. (I) Nomarski light micrograph (differential interference contrast) of cell cluster in (H).

During the first 7 d in medium containing epidermal growth factor (EGF) and fibroblast growth factor (FGF), Spoc cells undergo several rounds of division, begin to express GATA-4 (a mostly cardiac-specific transcription factor), and become clusters of floating round cardiac precursors from Spoc (CPS) cells with an increased diameter of 10–14 μm. The pattern of GATA-4 staining in these cells is unusual in that it is predominantly cytoplasmic, which is distinct from the expected nuclear staining of a transcription factor. Although rare, many transcription factors are well known to have a cytoplasmic phase. GATA-4 has been described similarly and in this case has been shown to move into the nucleus after the addition of the beta-adrenergic drug isoproterenol [24,25]. For these reasons, isoproterenol was added to a culture of CPS cells for 1 h, after which GATA-4 staining was observed in the nucleus of many of the cells (Figure 2). These cells go on to express other cardiac-specific markers, including cardiac troponin-T, Nkx-2.5, MLC-2v, and a cardiac L-type calcium channel, detected either by immunostaining or real-time PCR (Figure 3A–3G; real-time PCR data not shown). Although Nkx-2.5 may be present in vascular smooth muscle, neither alpha- nor beta-myosin is present in smooth muscle. Beating cardiomyocytes derived from Spoc cells express both alpha- and beta-myosin at day 28, as shown by staining with polyclonal antibodies against alpha- and beta-myosin (Figure S1). In later stages, connexin 43 is expressed in cell clusters (Figure 3H–3I). Even before they become adherent, some isolated cells begin to beat (Video S1).

### Sca-1 Separation of Spoc Cells

Further fractionation of Spoc cells by sorting for the Sca-1 marker (a cell-surface antigen found on murine hematopoietic stem cells [HSCs]) shows that the beating cells develop out of the Sca-1^−^ pool, which comprises 20%–40% of the total isolated cells. This is in contrast to recent studies in which an isolated Sca-1^+^ population from the heart homed to the heart after vascular injection and showed signs of cardiac differentiation [14,26]. When the skeletal muscle–derived Sca-1^+^ fraction of Spoc cells are plated under our conditions (see Materials and Methods) separately from the Sca-1^−^ fraction, the former cells rapidly adhere to the plate and, with the exception of a few putative contaminating Sca-1^−^ cells, do not develop into beating cells. With the Sca-1^+^ population removed, the remaining Sca-1^−^ population undergoes additional divisions before beginning to differentiate. Occasionally, a small, round beating cell with no organized sarcomere is seen after 3 d, but approximately 80% of the total population of cells differentiates into beating cells after a 7- to 10-d proliferative period. These Sca-1^−^–derived CPS cells remain in this immature state, i.e., round, loosely attached, and spontaneously beating, for more than 2 mo of culture. When a green fluorescent protein (GFP)–tagged Sca-1^−^ population of CPS cells is replated onto a nontagged adherent monolayer of the purified Sca-1^+^ cells, the fluorescent Sca-1^−^ cells adhere to the monolayer within 24 h, stretch out, and mature into beating cells. As the stretched-out GFP^+^ cells develop a sarcomeric structure, the GFP signal diminishes. This phenomenon, which is possibly due to a down-regulation of the beta-actin promoter-driven GFP, or to the exclusion of GFP protein from the developing sarcoplasm, may compromise the detection of GFP-tagged donor cells in the mouse infarct model.

### Spoc Cells Are Not Derived from Bone Marrow or Adipose Tissue

Spoc cells do not appear to be bone marrow cells sequestered in skeletal muscle, and because they are c-kit^−^, they are distinguished from the c-kit^+^ bone marrow cells and side population cells that have been used directly or indirectly in experiments to reconstitute infarcted heart [11,13]. When approximately 50 mg of whole marrow or dissociated total heart is co-cultured in permeable-membrane-separated compartments in a 1:1 ratio with Spoc cells, only the Spoc cells develop into beating cardiomyocytes. Thus, bone marrow and heart do not contain a cell population that is isolatable in this manner and phenotypically similar to Spoc cells.

We also performed experiments to determine whether Spoc cells are similar to adipocyte-derived stroma vascular fraction cells (SVFs), which have been derived from a mixture of murine inguinal and intrascapular fat pads [19]. Spoc cells were cultured in parallel with SVFs in our EGF/FGF–containing medium, as well as in methocult supplemented with beta-mercaptoethanol, erythropoietin, Interleukin (IL)–3, IL-6, and Stem Cell Factor, as outlined in [19], except that inguinal- and intrascapular-derived cells were kept separate from each other. In the supplemented methocult culture, Spoc cells developed into CPS cells, which subsequently fused into large (up to 1 mm in length) beating myocytes as described earlier [19]. We were not able to generate CPS cells, or any beating cells, from SVFs derived from inguinal fat alone. A result similar to culturing Spoc cells was observed with intrascapular-derived SVFs cultured in supplemented methocult, but only in cultures that had microscopic contamination with skeletal muscle fibers. Neither SVFs from the inguinal nor from the intrascapular fat pads yielded any beating cells when cultured under standard EGF/FGF Spoc cell conditions. Planat-Benard et al. [19] report an efficiency of conversion to cardiomyocytes of 0.02%–0.07%, whereas 1%–10% of total Spoc cells become beating cardiomyocytes. When pure Sca-1^−^ Spoc cells are plated, after the proliferation phase, approximately 80% of the cells are beating CPS cells.

Unlike the case with HSCs, no colony-forming units are generated when Spoc cells are cultured in methylcellulose in the presence of erythropoietin, IL-3, IL-6, and Stem Cell Factor (data not shown). To evaluate the ability of Spoc cells to reconstitute bone marrow, standard and competitive bone marrow transplantations with Spoc cells were performed. In four mice, 3 × 10^6^ bone marrow cells and 3 × 10^4^ GFP ^+^/Sca-1^−^ Spoc cells were injected into each of the lethally irradiated mice. All four mice survived; however, only a rare GFP^+^ donor–derived cell was seen in the peripheral blood or bone marrow (data not shown). The marrow of six lethally irradiated mice, injected with either 1.5 × 10^5^ Sca-1^−^ Spoc cells or 2 × 10^5^ Spoc cells unfractionated for Sca-1, could not be rescued, and all six mice died within 2 wk. Thus, because only a single HSC is required to reconstitute bone marrow, no HSCs are contained in 2 × 10^5^ Spoc cells.

### Transmission EM of CPS Cells as They Progress to Beating Cells

**Figure 4:**
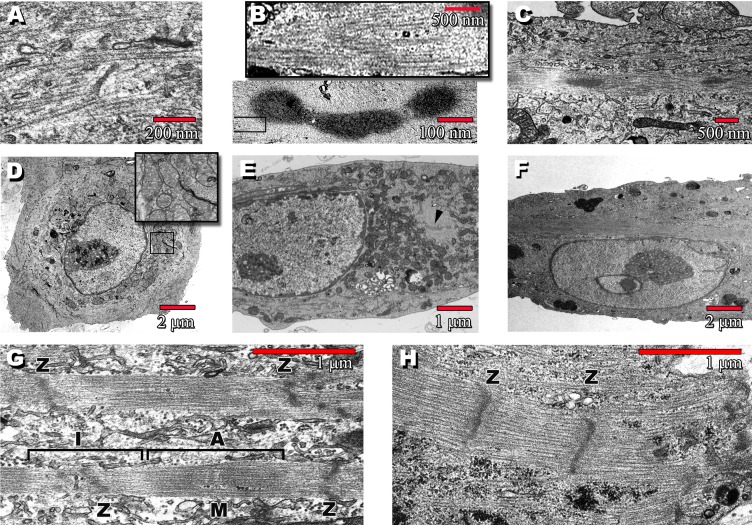
Transmission EMs Show the Post-Replating Progression of CPS Cells (A) Round, day 3 cells contain disordered myosin filaments. Some of these cells beat while still floating (see Video S1) and typically have APs as shown in Figure 6A. (B) Upper box is a blowup taken from lower panel, showing myosin filaments of characteristic 1.6-μm length radiating outward from dense body. (C) Day 14 cell with a single, central nucleus shows a stretching out of the dense bodies into an organizing sarcomere. (D) Day 3 round cells containing copious mitochondria (inset). (E) Elongated day 7 cell containing a dense body (arrowhead). (F) Uninucleate day 14 cell, same cell as in (C). (G) By day 56, a well-defined sarcomere is present, with identifiable A- and I-bands and M- and Z-lines. (H) Sarcomere from a fetal cardiomyocyte is shown for comparison.

Figure 4 shows the post-replating progression of CPS cells. Three days after the replating of floating cells (see Materials and Methods), when increasing numbers of CPS cells show rhythmic beating, EM shows round cells with large central nuclei surrounded by copious mitochondria and myosin thick filaments (Figure 4A and 4D). As the cells attach to the tissue culture plate, the central nucleus elongates and electron-dense structures are visible (Figure 4B; arrowhead in Figure 4E), with myosin filaments radiating outward (box/inset, Figure 4B). By 2 wk, the electron-dense bodies have begun to align with filaments coursing between them (Figure 4C). These structures are nearly identical to those seen in embryoid body–derived developing cardiomyocytes [27]. By 8 wk in culture, the electron-dense structures in EMs of beating cells have progressed to become the Z-lines of organized sarcomeres (Figure 4G). The beating cells also have one or two large, centrally located nuclei surrounded by mitochondria, distinguishing them from skeletal myotubes, which have many small, subsarcolemmal nuclei.

### Calcium Transients and Cardiac Action Potentials in CPS Cells

**Figure 5:**
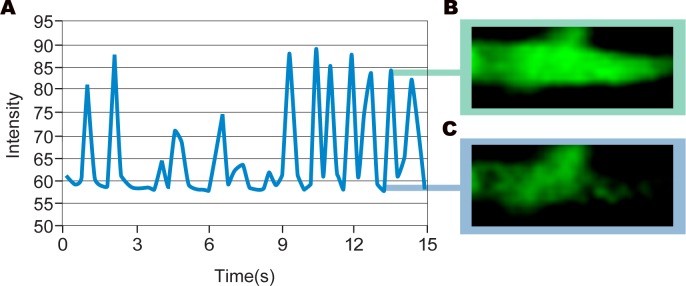
Measuring Calcium Transient Frequency Graphical representation of the calcium transient in a beating CPS cell–derived cardiomyocyte (A). Fluorescent intensity is proportional to the amount of calcium binding to fluo-3 dye upon release of calcium from the sarcoplasmic reticulum. Peak intensity (B) and baseline (C) are shown.

**Figure 6:**
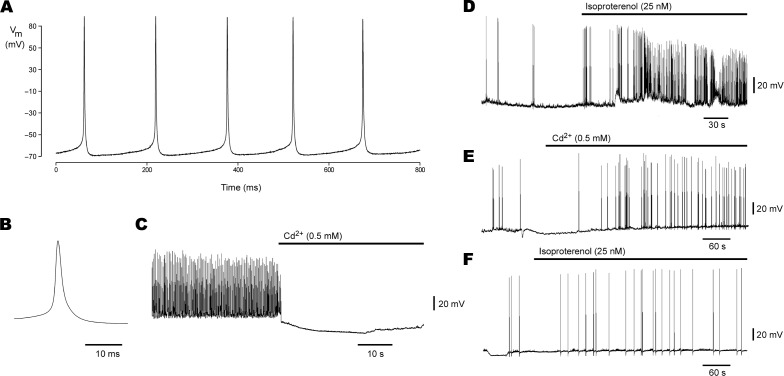
Whole-Cell Voltage Recordings from Spoc Cell–Derived Cardiomyocytes (A) Spontaneous AP firing in a nonbeating, teardrop-shaped cell. (B) Representative AP from recording in (A) on an expanded time scale; AP threshold is –60 mV. (C) Action potential firing in another cell is blocked upon bath perfusion with 0.5 mM cadmium chloride (horizontal bar). (D) Acceleration of AP firing upon perfusion with 25 nM isoproterenol (horizontal bar) is demonstrated, indicating the presence of adrenergic receptors on these cells. (E) Skeletal myotube APs, if present, differ in that their frequency is unaffected by Cd^++^. (F) Isoproterenol also does not affect skeletal muscle AP frequency.

As the CPS cells progress to spontaneously beating cardiomyocytes in vitro, the frequency of beating ranges from 1 to 8 Hz. Cells continue to beat even after culture for 3 mo. Despite the absence of beating initially, some clusters of cells adherent to an extracellular matrix display calcium transients detected by fluo-4 (4 μM concentration) visualized with confocal microscopy (Video S2). This suggests that the excitation portion of “excitation–contraction” develops before the contractile apparatus of the cell is mature enough to overcome the minimal resistance offered by an extracellular matrix. The round beating cells shown in Video S1 progress to the elliptical beating cells shown in Video S3 and subsequently to the more mature cardiomyocytes shown in Video S4. These last cells also display calcium transients detected through the use of fluo-3 (4 μM concentration) as shown in Video S5 and Figure 5. By day 14 after replating, 10% of the cells in a confluent dish beat spontaneously. The calcium transients indicate the existence of action potentials (APs), which have been characterized by patch recordings of single cells in culture (Figure 6). A variety of cardiac APs are observed in beating and nonbeating cells (Figure 6A and 6B), which both show a resting membrane potential of approximately −60 mV with a robust overshoot of 50–90 mV. The form and duration of the APs match the descriptions of adult murine cardiomyocyte APs, which lack the plateau phase seen in cardiomyocytes of other species [28].

The beating in Spoc cell–derived cardiomyocytes differs from the sporadic twitches that have been observed in skeletal muscle because the addition of 0.5 mM cadmium chloride, a non-specific blocker of L-type Ca^++^ and Na^+^ channels, abolishes the cardiac AP [29,30], while having no effect on skeletal myotubes (Figure 6C and 6E) [31,32]. Likewise, both beating and nonbeating cardiac cells have an intact adrenergic pathway [33,34,35], as shown by the increase in AP frequency with the addition of 25 nM isoproterenol (Figure 6D). The expected lack of effect on skeletal myotubes [36] is shown in Figure 6F. Uninucleate myoblasts with spontaneous calcium transients and APs under standard culture conditions have not been described [37], and even when they are contrived by arresting cell fusion, they retain the electrical activity characteristic of skeletal muscle cells, such as the lack of response to cadmium chloride [38].

### Transplantation of Spoc Cells into Murine Myocardial Infarction Models

**Figure 7:**
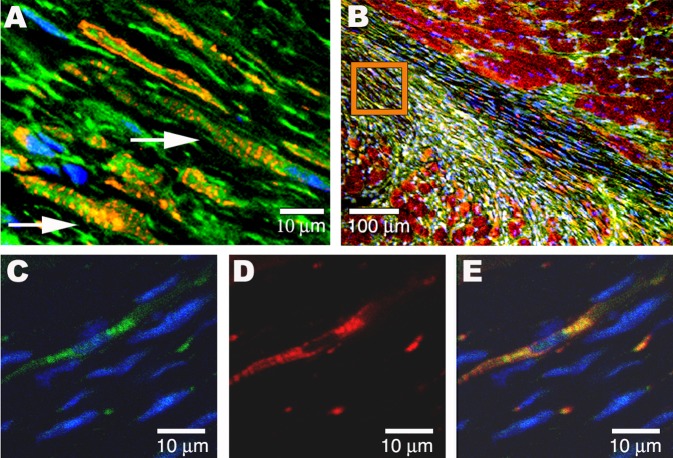
In Vivo Myocardial Infarction Transplantation Studies (A) The GFP-tagged Spoc cells (green), unfractionated for Sca-1, injected into the peripheral blood of a murine acute infarct model have developed after 14 wk into cardiomyocytes (arrows) within the infarct region. Donor Spoc cell–derived GFP cardiomyocytes are characterized by single central nuclei and striations staining for RLCP (red). (B) Longitudinal fresh-frozen tissue slice showing the region of infarct from which (A) was taken (orange box) and adjacent normal endogenous cardiac tissue (RLCP, red). (C) GFP^+^/Sca-1^−^ Spoc cells were injected into the peripheral circulation of a murine acute MI model and are detected in the infarct after 4 wk by expression of GFP (green). (D) RLCP (red) is also expressed. (E) Overlay of (C) and (D).

In order to determine whether Spoc cells engraft within a myocardial infarct (MI) and differentiate into mature cardiomyocytes, an acute infarct model in C57Bl/6J mice was created by ligation of the left coronary artery. We injected 1 × 10^5^ GFP^+^ total Spoc cells (unfractionated for Sca-1) into the peripheral circulation. After 14 wk, many donor-derived GFP^+^ cells had engrafted (Figure 7B). Of donor cells that had migrated to the infarct, 8% (63/782) had developed into cardiomyocytes (arrows, Figure 7A) displaying a phosphorylated cardiac myosin regulatory light chain (RLCP), shown by co-labeling with an antibody against GFP (green) and RLCP (red) [39]. All samples were scanned on green and red channels simultaneously. Cells were considered GFP^+^ only if their cytoplasm fluoresced green and not red. This prevents mistaking the anti–GFP signal (green) in Figure 7A for autofluorescence. Autofluorescence in an infarct will characteristically “bleed” through both green and red channels in the absence of exogenous fluorescent markers, causing all autofluorescence to show up as a generalized yellow, i.e., the combination of red and green [40]. In this case, only the myosin striations, which are co-stained, appear as both red and green, serving as an internal control for the technique. No labeled cells, differentiated or undifferentiated, were observed in the normal portion of the infarcted heart (data not shown). These findings suggest that Spoc cells either actively home to or are passively delivered to an area of cardiac damage where they begin to differentiate into cardiomyocytes, as observed in vitro. In order to determine if Sca-1^−^ Spoc cells engraft and differentiate within a MI as efficiently as Spoc cells unfractionated for Sca-1, the same acute anterior MI model described earlier was used. When 1 × 10^5^ Sca-1^−^/GFP^+^ donor cells were injected via the tail vein immediately after infarction, a low level of engraftment occurred with only an occasional donor-derived cardiomyocyte found at the periphery of the infarct zone (Figure 7C–7E). This finding is consistent with our in vitro findings described earlier, in which purified Sca-1^−^ cells remained immature, loosely attached, round beating cells for months until co-culture with a feeder layer of immobile Sca-1^+^ cells allowed the Sca-1^−^ fraction to adhere and mature.

To evaluate for a similar effect in an older infarct, the same number of Spoc cells, unfractionated for Sca-1, was injected via the tail vein into two mice (8 and 14 wk after MI). Two weeks after injection, the heart of the 8-wk-old infarct model showed co-localization of GFP and GATA-4 in 3% (4/136) of donor cells that migrated to the peripheral region of the infarct. Five weeks after injection into the 14-wk-old infarct model, an increased number of GFP^+^/RLCP^+^ cells (7/102 donor-derived cells) in the infarct region were evident (data not shown). Spoc cells that were partially differentiated by culturing for 7 d were injected into the hearts and tail veins of three mice with acute infarcts. No labeled cells were identified in the hearts at 7 d and 1 mo later, compared to two control mice injected with saline at the time of infarct (data not shown). This suggests that the undifferentiated cells more easily actively home to or are delivered passively to the heart.

**Figure 8:**
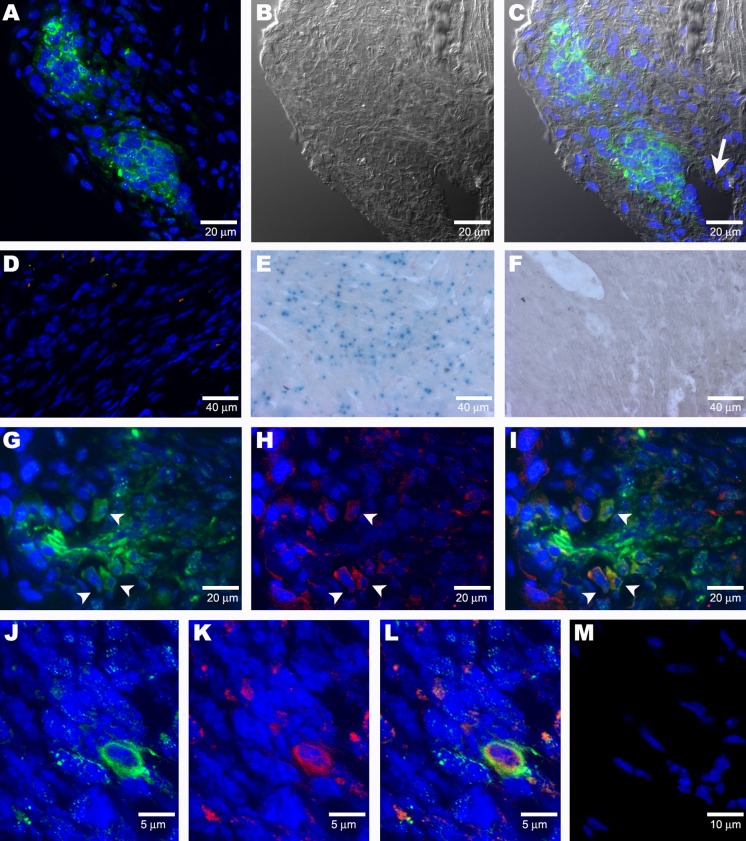
Cre Expressor/Beta-Galactosidase Reporter Myocardial Infarction Studies (A) Nests of Cre^+^ cells (green) are detected 1 wk after tail-vein injection into an acute infarct model. (B) Nomarski image of (A). (C) Merged image of (A) and (B). The clusters are located near a blood vessel (arrow). (D) Infarcted tissue in a control MI model (infarction surgery but no donor-cell injection) showing a lack of staining for Cre (no green) and GATA-4 (no red). (E) Control X-gal staining of ROSA mouse heart. (F) In a sequential series of tissue sections, odd-numbered sections were immunostained for Cre, yielding the results seen in (A). These two clusters of cells were seen on five sections (sections 1, 3, 5, 7, and 9). Even-numbered sections (sections 2, 4, 6, and 8) were stained for X-gal. No X-gal^+^ cells were found. One slide was immunostained, showing the Cre^+^ cells present. This slide was then stained for X-gal and was found to be X-gal^−^. The lack of X-gal staining of the serial sections indicates that at 1 wk no fusion of donor and host cells has occurred in the infarct. (G) Cluster of Cre^+^ donor cells detected in infarcted heart tissue of a 1-wk-old acute infarct model. Arrowheads indicate cells that also express GATA-4, as shown in (H). (H) GATA-4 (red) is mostly present in some cells in the margin of the cluster. Arrowheads indicate cells that also express Cre, as shown in (G) (I) Merged image showing co-localization (arrowheads) of Cre (green) with GATA-4 (red) in some cells of the cluster of Cre^+^ cells. (J–L) Co-staining of donor cells with anti–Cre antibody (green) and MSC 21 (red) is apparent after 7 d in an acute infarct model. (M) There is a lack of staining with MSC 21 (no red) in the infracted tissue of mice that have not received Spoc cell injections.

We used a Cre-recombinase (Cre) expressor/beta-galactosidase reporter system to rule out donor stem-cell fusion with host cardiomyocytes as an explanation for apparent in vivo differentiation. In the event of fusion, Cre from the donor cells will excise the floxed stop codon present in the host DNA and thus de-repress the beta-galactosidase reporter gene. Within hours of producing an acute infarct by surgical ligation of the left coronary artery in R26Rh reporter mice, tail-vein injection of 3 × 10^5^ Cre^+^ Spoc cells from an EIIa promoter/Cre donor mouse was performed. Cre^+^ donor cells are seen in the heart as early as 2 d post infarct (data not shown). By day 7, isolated nests of donor cells that have not fused with host cells can be detected, as shown by the lack of X-gal staining in Cre^+^ cells (Figure 8A–8F). Also by day 7, GATA-4 is co-expressed with Cre in some of the cells in these clusters (Figure 8G–8I). In order to further isolate and track donor Spoc cells, a monoclonal antibody library was generated by inoculating a rat with live Spoc cells via injection into the peripheral circulation. The library was screened for the ability to detect cell-surface antigens on total Spoc cells. One such antibody that has been generated (MSC 21) detects live Spoc cells in culture and can be used to isolate a positive and negative subpopulation. MACS separation using MSC 21 gives two populations, with the 21^+^ subset containing cells that develop into beating cardiomyocytes (Video S6). In the Cre donor/R26Rh reporter infarct mice, some of the injected Cre^+^ cells are co-stained by MSC 21, further supporting the donor origin of these transplanted cells (Figure 8J–8L). MSC 21 does not detect cells in normal controls or infarcted heart (Figure 8M).

### Concluding Remarks

Our isolation and description of what we call Spoc cells was initially surprising to us in that a few simple variations in the usual technique of skeletal muscle cell culture yielded a novel finding. These adaptations included avoiding trypsin, substituting FGF and EGF for commonly used complex supplements like chick embryo extract, and discarding adherent cells while continuing to culture the nonadherent cells. The observation of isolated nonadherent beating cells in a skeletal muscle culture has probably been observed previously by others in the course of experimenting with skeletal muscle culture conditions but has anecdotally been considered to be some variant of primitive skeletal muscle cells and never been carefully investigated . This report is, to our knowledge, the first time that the phenotype of these cells has been methodically studied with single-cell patch recordings, Ca^++^ transient recordings, EM, longitudinal video microscopy, and immunostaining to detect the presence of skeletal and cardiac histologic markers. The absence of skeletal and satellite cell markers in the face of the histologic and physiologic evidence of the cardiac phenotype supports our conclusion that these cells are closer to true cardiac myocytes than to some variant of skeletal muscle cells. The homing of these freshly isolated cells to the injured heart and their subsequent differentiation into a cardiac phenotype in vivo that matches the in vitro experiments is corroborating evidence.

Recently, three independent groups have isolated a stem-cell population from the heart. In two studies, the stem cells were cultured extensively [15,20] before reintroduction directly into the heart, whereas in the other study, freshly isolated cells were injected into the vascular circulation [14]. Beltrami et al. [15] did not observe beating cells in vitro, whereas the cells isolated by Oh et al. [14] required treatment with the demethylating agent 5-azacytidine to produce beating cells, similar to the treatment that was previously used to generate beating cell lines from bone marrow stromal cells [41]. Messina et al. [20] used cardiotropin to treat round cells released from a 3-wk explant culture, generating a sphere of cells that remain primitive and proliferating on the inside while they differentiate into beating cardiac myocytes at the margins. They observed that this growth pattern is reminiscent of neurospheres. All three cardiac-derived populations appear to be distinct from one another with respect to parameters such as the presence of c-kit or the ability to beat in vitro; however, the diverse conditions make direct comparison difficult.

Spoc cells do not emerge from cardiac tissue grown in parallel with the skeletal cultures. However, freshly isolated Spoc cells are observed in infarcted heart within 2 d of their systemic injection into a murine model of acute MI. Within 7 d of systemic injection, Spoc cells form a sphere-like body at the borders of the infarction near blood vessels. The Cre signal is strongly present in the center of these spheres but diminished at the borders where the GATA-4 signal emerges. This is likely due to EIIa promoter suppression in the differentiating cells. This pattern of the more proliferative cells in the center and the differentiating cells at the borders is reminiscent of neurospheres or the in vitro culture of cardiac-derived stem cells [20]. By 3 mo, many more of the cells are distributed throughout the border areas of the infarct and can be detected with an antibody to ventricular RLCP.

The pattern of GATA-4 staining in early Spoc cells is particularly interesting in its unusual cytoplasmic distribution, but the shift to a nuclear distribution after the addition of the beta-adrenergic agonist isoproterenol to the cell culture illustrates the staining specificity. The importance of beta-adrenergic stimulation to the processes of differentiation and cardiac myocyte hypertrophy makes these cells particularly useful to study the Akt–GSK3 beta regulation of nuclear-expressed GATA-4 [25] as well as its beta-adrenergic–mediated interaction with the calcineurin pathway [24].

Although the beating cells in vitro contract asynchronously, local regions of confluent plates appear to beat at distinct rates. This fact, coupled with the late expression of connexin 43 in vitro (see Figure 3H), suggests the potential for coordinated beating, a requirement for efficient cardiac contraction. Although Spoc cells appear more efficient at homing to the heart in an acute MI model, their ability to differentiate in vivo at the border of an old infarct may aid research focused on chronic heart failure or dilated cardiomyopathy. Generation of cardiomyocytes from Spoc cells derived from transgenic models may be an important tool in studying cardiac development, because the progression from undifferentiated cells to mature cardiomyocytes may be observed in vitro.

Why these cardiac stem cells are sequestered in skeletal muscle and why clinically significant numbers of them are not normally recruited to salvage an infarcted heart remains a mystery. Perhaps additional cells or factors present in skeletal muscle suppress the cardiac differentiation of Spoc cells in situ or inhibit migration. Certainly, Spoc cells do not differentiate into cardiac cells while they reside in skeletal muscle. Along these lines, fractionation of Spoc cells based on Sca-1 greatly enriches for a population of cells in the Sca-1^−^ fraction that in vitro become beating cardiomyocytes; however, engraftment of these Sca-1^−^ cells in MI models seems less efficient than that of unfractionated Spoc cells. This is consistent with the increased magnitude and kinetics of cardiomyocyte differentiation observed when the Sca-1^−^ fraction is plated over a monolayer of the Sca-1^+^ fraction. Thus, there is likely a role for the Sca-1^+^ fraction in engraftment efficiency through any number of mechanisms, including migration, tropism, and entrapment. It remains to be determined whether Spoc-cell transplants contribute to mouse survival or electrically couple to endogenous cardiac myocytes. There is evidence, however, that Spoc cells survive more than 3 mo post transplant, during which time they mature into striated cardiomyocytes.

## Materials and Methods

### 

#### Isolation of Spoc cells

To isolate cardiomyocyte precursor cells from adult mouse skeletal muscle, skeletal muscle tissue from the hind legs of 6- to 14-wk-old male C57Bl/6J mice is cut into small pieces and digested with collagenase type-2 (5 mg/ml) for 45 min at 37 ^o^C, and then for another 45 min at 37 ^o^C in a shaking rotator. The digested tissue is cleared of cell debris and other undigested tissue fragments by passage first through 100 μm and then through 40 μm filters. Cells are incubated in DMEM/F12 medium containing 5% FBS for 2 h at 37 ^o^C. The cell suspension is then centrifuged at 1,500 RPM for 15 min. The pellet consists mostly of small cells, 4–10 μm in diameter. These cells are dispersed by trituration and are then sorted using anti–Sca-1 antibody on Miltenyi Biotech (Bergisch Gladbach, Germany) magnetic columns so that the Sca-1^−^ cells from total hind leg muscles of five to eight mice are passed sequentially through three to four columns to obtain a high degree of Sca-1^−^ purity (>95% by fluorescent staining).

Cells are plated at a density of approximately 10^5^ cells/cm^2^ in regular tissue culture dishes in 50:50 DMEM/F12 supplemented with 5% FBS, 10 ng/ml each of human EGF and bFGF (Peprotech, Rocky Hill, New Jersey, United States), insulin, transferrin, selenium, ethanolamine (ITS-X; Invitrogen, Carlsbad, California, United States ), and antibiotics (gentamicin and fungizone). Within 24 h, it is clear that the Spoc cells eluted in the Sca-1^−^ fraction consist of mostly nonadherent round cells, whereas the Sca-1^+^ fraction consists of mainly adherent cells. Within 48–72 h, the Sca-1^−^ culture consists of a floating population of round cells and scant adherent fibroblasts. The round cells enlarge as they divide in this medium, undergoing a few rounds of cell division. The floating population of round cell clusters is gently collected after 7 d of growth in complete growth medium and replated in the same medium, minus EGF and bFGF. The cells, which have enlarged to 10–14 μm in diameter, are by now GATA-4 positive. Spontaneously beating cells are visible while they are still floating or loosely attached, but the number of beating cells increases within a few days of replating as the cells elongate and attach to the floor of the tissue culture plate. The beating cells do not undergo any more cell divisions and can be maintained in this medium for several weeks, displaying a spontaneous beating phenotype.

Alternatively, the total population of Spoc cells, unfractionated for Sca-1, can be plated in growth-factor-containing medium for 3–5 d and then the nonadherent cells removed carefully and replated without growth factors. This will result in a greater population of what will become adherent cells in the replated culture. This allows the nonadherent Spoc cells to adhere and stretch out into more mature beating cardiomyocytes. The isolation and differentiation of Spoc cells from skeletal muscle is extremely reproducible and has been performed successfully in our laboratory hundreds of times. These cells have been used for characterization, as described in the text, at different times after the initiation of the culture.

#### Transmission EM

For routine transmission EM, cells were fixed in situ on petri dishes with 1.25% glutaraldehyde in 0.1M cacodylate buffer containing 1% cadmium chloride at 4 ^o^C for 2 h. After fixation, cells were washed three times in Sabatini's solution (0.1 M cacodylate buffer containing 6.8% sucrose), and post-fixed with 1% osmium tetroxide in cacodylate buffer for 1 h. After three washes in Sabatini's solution, samples were dehydrated in alcohol and embedded in Scipoxy 812 (Energy Beam Sciences. Agawarm, Massachusetts, United States). Polymerization was carried out at 37 ^o^C for 24 h and then at 60 ^o^C overnight. Ultra-thin sections were cut with a Leica Ultracut UCT ultramicrotome (Leica, Wetzslar, Germany), stained with uranyl acetate and Reynolds lead citrate, and examined with a JEOL 1200 CXII transmission electron microscope (JEOL, Peabody, Massachusetts, United States).

#### Confocal imaging and detection of calcium transients

The images were collected with a Zeiss LSM-510 laser scanning confocal system and a Zeiss C-Apochromat 63x objective (1.2 NA). Fluo-3 and fluo-4 were excited at 488 nm with an argon laser, and the emission light was collected using an LP 505 filter. The pinhole was adjusted to produce a 5-μm slice to minimize axial movements with contraction that may affect viewing the calcium transients. All transmitted light images were collected simultaneously using a transmitted light detector in conjunction with the 488-nm excitation light. Data depth for the images was 8 bit. The size of the images varied from 512 × 512 pixels to 128 × 128.

#### Whole-cell clamp recordings

Current clamp recordings were carried out using the tight-seal whole-cell patch technique at room temperature in Tyrode solution containing 136 mM sodium chloride, 5.4 mM potassium chloride, 1 mM magnesium chloride, 1.8 mM cadmium chloride, 0.33 mM sodium phosphate monobasic monohydrate, 10 mM glucose, and 10 mM HEPES (adjusted to pH 7.4 with sodium hydroxide). The pipette solution contained 20 mM potassium chloride, 110 mM potassium aspartate, 1 mM magnesium chloride, 10 mM HEPES, 5 mM EGTA, 0.1 mM GTP, and 5 mM Mg^2+^/ATP (adjusted to pH 7.2 with potassium hydroxide). Voltages were filtered at 2 kHz (–3 dB; four-pole, low-pass Bessel filter). The resting membrane potential upon breaking in was –39.8 ± 1.6 mV (*n* = 9), but it generally improved by 10 mV or more. In some cases, cells were hyperpolarized slightly to AP threshold.

#### Immunostaining

Cells were fixed in either 4% paraformaldehyde at 4 ^o^C, neutral buffered 10% formalin at 4 ^o^C, or acetone at –20 ^o^C for 10 min and then washed in PBS for a total of 15 min (three times). Cells were permeabilized with 0.2% Triton X-100 for 10 min. Blocking was performed for 30 min at room temperature with either 3% BSA in PBS, 5% goat serum in PBS, or 10% goat serum in PBS. Incubation with primary antibody was performed at 4 ^o^C overnight. Cells were then washed for a total of 15 min (three times) in 1X PBS. Incubation with secondary antibody was done at room temperature for 1 h. Afterwards, cells were washed three times with PBS and mounted in a fluorescent mounting medium with 4′,6-diamidino-2-phenylindole (DAPI) (Vector Laboratories, Burlingame, California, United States). A cover slip was then placed over the sample. Images of cells were obtained with a laser scanning confocal fluorescence microscope (Leica TCS-4D DMIRBE) equipped with argon and argon–krypton laser sources. Excitation wavelengths of 365 nm (DAPI), 488 nm (FITC), and 568 nm (rhodamine) were used to generate fluorescence emissions in blue, green, and red, respectively. The primary antibodies used were against MyoD (Novocastra Laboratories, Newcastle-upon-Tyne, United Kingdom), GFP (Abcam, Cambridge, United Kingdom), cardiac L-type channel (United States Biological, Swampscott, Massachusetts, United States), Sca-1 (Cedarlane Laboratories, Hornby, Ontario, Canada), and CD34 and CD45 (PharMingen, San Diego, California, United States). GATA-4, Myf-5, Myogenin, connexin 43, Nkx-2.5 Pax-3/7, c-met, and c-kit antibodies were from Santa Cruz Biotechnology (Santa Cruz, California, United States). The antibody to RLCP was produced in our laboratory and has previously been described [39]. Alpha-myosin antibody has been produced and characterized by our laboratory and is unpublished. Beta-myosin antibody was produced by our laboratory and previously published [42]. Antibody MSC 21 was generated after peripheral blood injection of Spoc cells into rats (performed by Antibody Solutions, Palo Alto, California, United States). Figure S1 was taken using a Zeiss Axiovert 200M microscope in conjunction with Compix (Lake Oswego, Oregon, United States) deconvolution software. To perform GATA-4 sublocalization studies, CPS cells in culture dishes (day 10) were incubated with 20 μM isoproterenol at 37 ^o^C for 1 h. Cells were then collected by trituration, cytospins were made, and cells were stained for GATA-4, as described earlier.

#### Bone marrow transplantation studies

Eight 1-wk-old C57Bl/6J mice were irradiated with one total dose of 850 cGy. Four hours after irradiation, mice were injected via the tail vein with either 1.5 × 10^5^ GFP^+^/Sca-1^−^ Spoc cells or 2 × 10^5^ unfractionated GFP^+^/Spoc cells. A competitive assay was performed by injecting mice with 3 × 10^6^ whole bone marrow cells and 3 × 10^4^ GFP^+^/Sca-1^−^ Spoc cells. Mice were evaluated for GFP^+^ cells in the peripheral blood and bone marrow.

#### MI studies

Sixteen 1-wk-old male C57Bl/6J mice were administered Avertin (1.25%, tribromoethanol) at 0.015 ml/g body weight or pentobarbital diluted to 0.5% and administered at 100 mg/kg IP. Animals were intubated by direct visualization, and ventilation was performed. Once anesthetized, hair in the surgical field was removed, and a thoracotomy was performed. Heart was visualized after retractors were used to enlarge the incision. The left anterior descending artery was permanently ligated using 5-O silk suture. The chest was closed with sutures. For acute infarct models, mice were monitored for recovery from anesthesia and then injected (2 h post surgery) with 1 × 10^5^ C57Bl/6J Spoc cells (either fractionated or unfractionated) via the tail vein. Mice were sacrificed at 14 wk; the hearts were excised and fresh frozen sections made. For chronic infarct models, infarction protocol was performed as described earlier. The mice were maintained for either 8 or 14 wk before injection of 1 × 10^5^ Spoc cells into the tail vein. Mice were sacrificed at either 2 or 5 wk post injection, and the hearts were harvested for frozen sections. For Cre expressor/beta-galactosidase reporter MI studies, the infarct procedures described earlier were performed in 16-wk-old R26Rh mice. We obtained 3 × 10^5^ donor cells from 8-wk-old EIIa/Cre mice and injected the cells via the tail vein on the day of surgery.

Animal studies were created under National Institutes of Health protocols 2-MC-31(R) and 1-CB-2, in accordance with the guidelines set forth by the National Heart, Lung, and Blood Institute Animal Care and Use Committee.

## Supporting Information

Figure S1 Day 28 Spoc-Derived Cardiomyocytes Express Alpha- and Beta-Myosin (A) Day 28 Spoc cell–derived cardiomyocytes express alpha-myosin, as shown by immunostaining using anti–alpha-myosin antibody.
(B) Beta-myosin is also present in these cells.
(531 KB TIF).

Video S1 Round Beating Cell in Culture Video microscopy of round, spontaneously beating cell about 10 d from isolation (diameter = 15 μm), at approximately the stage shown in Figure 4D.
(5.1 MB MPG).

Video S2 Calcium Transients in Beating and Nonbeating CPS Cells Calcium transients are present in a cluster of day 14 beating cells in culture as detected by fluo-4 fluorescent dye. Only some of the cells with detectable calcium transients are visibly contracting, which shows an uncoupling of excitation–contraction, presumably because of an immature contractile apparatus or decreased movement caused by a dense extracellular matrix.
(7.0 MB MPG).

Video S3 More Mature Beating Cells Appear Elliptical The round beating cell shown in Video S1 has progressed by day 14 after replating to an elliptical cell (length = 30 μm) that continues to display small contractions as shown by video microscopy.
(7.4 MB MPG).

Video S4 Mature Beating CPS Cell–Derived Cardiac Myocyte in Culture Beating cell in culture (length = 55 μm). Spontaneous beating is continuous (frequency 1–6 Hz) and appears to be indefinite. Cells kept at room temperature have been noted to beat continuously for at least 3 h, and 3-mo-old cultures contain beating cells.
(7.6 MB MPG).

Video S5 Beating Cells Exhibit Calcium Transients Calcium transients are seen in a day 21 beating cell, as detected by fluo-3 fluorescent dye. Flashes indicate binding of calcium to fluo-3 upon release of calcium from the sarcoplasmic reticulum.
(3.5 MB MPG).

Video S6 MSC 21 Selects for a Subset of Cells That Develop into Beating Cardiomyocytes A cluster of beating cardiomyocytes derived from MACS-separated MSC 21^+^ cells is shown here after 14 d in culture.
(9.2 MB AVI).

## Abbreviations

AP: action potential
CPS: cardiac precursors from Spoc
Cre: Cre recombinase
DAPI: 4′,6-diamidino-2-phenylindole
EGF: epidermal growth factor
EM: electron microscopy
FGF: fibroblast growth factor
GFP: green fluorescent protein
HSC: hematopoietic stem cell
IL: interleukin
MI: myocardial infarct
RLCP: phosphorylated cardiac myosin regulatory light chain
Spoc: skeletal-based precursor of cardiomyocytes
SVF: stroma vascular fraction cell
